# Impaired 26S Proteasome Assembly Precedes Neuronal Loss in Mutant UBQLN2 Rats

**DOI:** 10.3390/ijms22094319

**Published:** 2021-04-21

**Authors:** Wenjuan Zhang, Bo Huang, Limo Gao, Cao Huang

**Affiliations:** Department of Pathology, Anatomy & Cell Biology, Thomas Jefferson University, 1020 Locust Street, Philadelphia, PA 19107, USA; Wenjuan.zhang@jefferson.edu (W.Z.); langlang@sxmu.edu (B.H.); gaolimo@126.com (L.G.)

**Keywords:** ALS, FTD, UBQLN2, proteasome, protein aggregation, rat

## Abstract

Proteasomal dysfunction is known to be associated with amyotrophic lateral sclerosis and frontotemporal degeneration (ALS/FTD). Our previous reports have shown that a mutant form of ubiquilin-2 (UBQLN2) linked to ALS/FTD leads to neurodegeneration accompanied by accumulations of the proteasome subunit Rpt1 in transgenic rats, but the precise pathogenic mechanisms of how this mutation impairs the proteasome remains to be elucidated. Here, we reveal that this UBQLN2 mutation in rats disrupted the proteasome integrity prior to neurodegeneration, that it dissociated the 26S proteasome in vitro, and that its depletion did not affect 26S proteasome assembly. During both disease progression and in an age-dependent manner, we found that proteasome subunits were translocated to the nucleus, including both of the 20S core particles (PSMA1 and PSMB7) and the 19S regulatory particles (Rpt1 and Rpn1), suggesting that defective proteasome function may result from the proteasome-subunit mislocalization. Taken together, the present data demonstrate that impaired proteasome assembly is an early event in the pathogenesis of UBQLN2-associated neurodegeneration in mutant UBQLN2 rats.

## 1. Introduction

Protein aggregation is one of the core features in both ubiquilin-2 (UBQLN2)-associated amyotrophic lateral sclerosis and frontotemporal degeneration (ALS/FTD) [1,2,3], a phenomenon that has been well replicated in transgenic rodents [4,5,6,7,8]. UBQLN2, a member of the UBQLN family, is involved in protein degradation via autophagy and the ubiquitin–proteasome pathway [9,10,11,12].

Pathogenic gene mutations in UBQLN2 are also known to lead to ALS and FTD in human patients [1,13]. As UBQLN2 interacts with microtubule-associated protein 1A/B-light chain 3 (LC3) via its ubiquitin-associated domain to regulate the formation of autophagic vacuoles [14]. It is interesting that recent reports implicate ALS/FTD-linked UBQLN2 mutations in proteasome dysfunction [15,16] and compromised autophagy in transgenic rodents with UBQLN2 mutations [4,5,6]. Together, these reports suggest that UBQLN2 mutations impair both autophagy and proteasome pathways.

Proteasomes are important for protein homeostasis and degrading aberrant or old proteins and damaged organelles [17]. Compared to the partial proteasome 20S, fully assembled proteasomes (the 26S: containing a 20S core particle with either one or two 19S regulatory particles) are more important for maintaining protein homeostasis, and their dysfunction is closely related to neurodegeneration [18,19,20]. While the 20S particle has all the proteases of a proteasome, the 19S particle has no protease activity but is necessary for the recognition/input of complex substrates [18,19,20]. Similarly, unstructured peptides can be degraded by the 20S particle alone, but complex substrates must first be unfolded by the 19S particle before being processed by the 20S particle for degradation [18,19,20].

The UBQLN2 gene is an X-linked, and the protein has an N-terminal ubiquitin-like domain and a C-terminal ubiquitin-associated (UBA) domain [21,22]. Both the UBL and the UBA domains participate in the interaction with proteasomes and are involved in autophagic protein degradation [14,23]. Via these domains, UBQLN2 shuttles ubiquitinated-proteins to proteasomes for degradation [24]. In transgenic mice, the accumulations of several proteasome subunits were caused by mutant UBQLN2 and these colocalized with UBQLN2 positive aggregates [4]. The depletion of the 26S proteasome was reported to cause neurodegeneration in mice [25], suggesting that 26S proteasome is necessary for normal neuronal survival. Osaka et al. reported that ALS/FTD-linked UBQLN2 mutations led to the in vitro accumulation of ubiquitinated-proteins in Neuro2a cells [15], again implicating UBQLN2 mutations in proteasome impairment. We similarly reported that mutant UBQLN2 transgenic rats accumulated both the autophagy substrate sequestosome-1/p62 and the proteasome regulatory subunit 7 (PSMC2, also called Rpt1) [26]. All these in vitro and in vivo studies suggest that proteasome impairments and gain-of-toxic function are caused by ALS/FTD-linked UBQLN2 mutations. However, the precise mechanisms by which UBQLN2 mutations affect proteasome function are still not known. Here, we examined the effect of an ALS/FTD-linked UBQLN2 mutant on the proteasome integrity and found that impaired proteasome assembly occurs prior to neurodegeneration in mutant UBQLN2 transgenic rats and that no proteasome alterations were observed in UBQLN2-depleted rats.

## 2. Results

### 2.1. Proteasome Impairment in Mutant UBQLN2 Transgenic Rats

**Figure 1:**
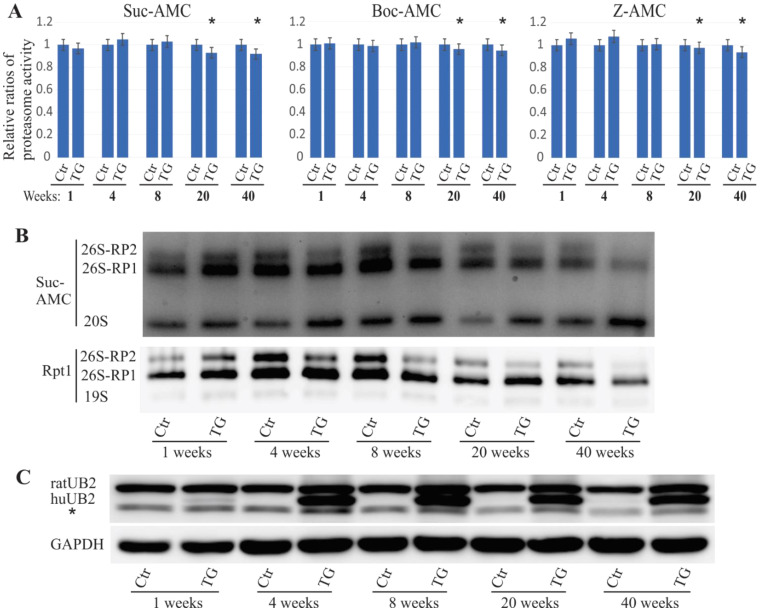
Impaired proteasome assembly in mutant UBQLN2 rats. (**A**). The overall activity of proteasomes was measured using sensitive fluorogenic substrates (Boc-AMC, Suc-AMC, or Z-AMC). The data are reported as the mean ± standard deviation (*N* = 4), * *p* < 0.05. (**B**). The in-gel assay (Suc-AMC) revealed that 26S proteasome assembly was impaired in mutant UBQLN2 rats, and in the immunoblot analysis using the Rpt1 antibody and 3–12% NativePAGE. Rat forebrains was homogenized in native protein lysis buffer, and 50 µg of total protein lysates were examined for the analysis. 26S-RP1/RP2: 26S proteasome containing either one or two regulatory subunits. (**C**). Immunoblot by SDS-PAGE showing UBQLN2 expression levels. Of total protein lysates 10 µg were assessed and the GAPDH signal was used as the loading control. RatUB2: endogenous UBQLN2; huUB2: human UBQLN2.

We previously reported that an ALS/FTD-linked UBQLN2 mutation led to both neuronal death [6] and the accumulation of proteasome subunit Rpt1 in rats [26]. In these rats, embryonic transgene expression was suppressed using 50 µg/mL doxycycline (DOX) in drinking water, and the DOX was deprived from the drinking water after birth. To study the effect of mutant UBQLN2 on proteasome function, we examined both proteasome activity and assembly in these rats. The overall proteasome activity was measured using proteasome assay buffer with either Boc-LRR-AMC substrate, Suc-LLVY-AMC substrate, or Z-LLE-AMC substrate for trypsin-, chymotrypsin-, or caspase-like proteasome activity, respectively. Based on the protein-lysate samples, we observed that overall frontal-cortex proteasome activity was unaltered until 20 weeks of age, but then worsened to the age of 40 weeks, with identical results obtained for the three different substrates (Figure 1A). These in vivo results indicate that proteasome activity was impaired during the late stages of the disease caused by mutant UBQLN2 expression in rats. Proteasome integrity was assessed using in-gel assay with the Suc-AMC substrate, followed by immunoblot assessment using the Rpt1 antibody. We found that 26S proteasomes that included a single regulatory subunit (26S-RP1) and two regulatory subunits (26S-RP2) were decreased in mutant UBQLN2 rats as early as four weeks of age and further deteriorated with age (Figure 1B,C). As previously described, mutant UBQLN2 has been shown to cause neurodegeneration in rats by 18 weeks of age. The present findings suggest that proteasome impairment precedes neuronal loss in mutant UBQLN2 rats.

### 2.2. No Observed Alterations of Proteasome in UBQLN2-Depleted Rats

**Figure 2:**
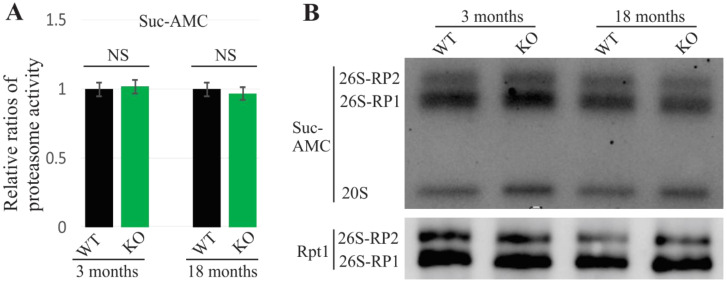
No observed proteasomal impairment in UBQLN2 knock-out rats. (**A**). The proteasome activity was measured using the Suc-AMC substrate. The data are reported as the mean ± standard deviation (*N* = 4). NS: no significant difference. (**B**). The in-gel assay revealed that 26S proteasome assembly was unchanged in UBQLN2 knock-out (KO) rats. Rat forebrains were homogenized in native protein lysis buffer and 50 µg of total protein lysates were assessed.

The complete loss of UBQLN2 does not exhibit any behavioral or pathological alterations in rats at 10 months of age [6]. To examine the effect of UBQLN2 loss on proteasome function, a total proteasome activity assessment and an in-gel assay assessment were both performed using UBQLN2-knockout rats. No overt changes were detected either in proteasome activity or in proteasome integrity, even at an 18-month time point (Figure 2). Together with the results from the mutant UBQLN2 rats, these findings are consistent with the idea that mutant UBQLN2 causes neurodegeneration via a gain-of-toxicity function.

### 2.3. Mislocalized Proteasome Subunits in Mutant UBQLN2 Transgenic Rats

**Figure 3:**
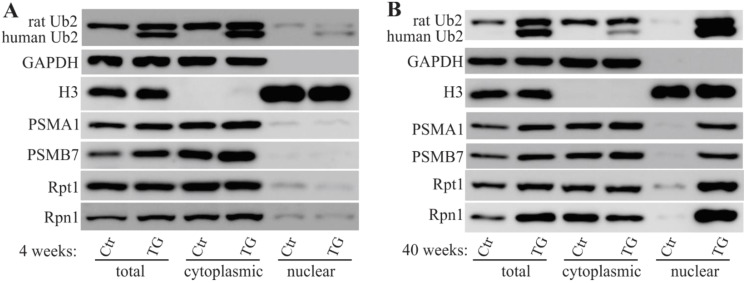
Altered proteasome subunit expression in mutant UBQLN2 rats. Cell fractionation revealed the expressions of proteasome subunits. Rat forebrains were dissected from mutant UBQLN2 transgenic (TG) and non-transgenic (Ctr) rats and tissue lysates were fractionated into nuclear and cytoplasmic components. The nucleus marker (histone 3: H3) and the cytoplasmic marker (GAPDH) were used to determine fractionation effectiveness. Human UBQLN2 and rat Ubqln2 (Ub2) were detected as two separate bands. The proteasome subunits PSMA1, PSMB7, Rpt1, and Rpn1 were examined in 4-week-old rats (**A**) and in 40-week-old rats (**B**).

**Figure 4:**
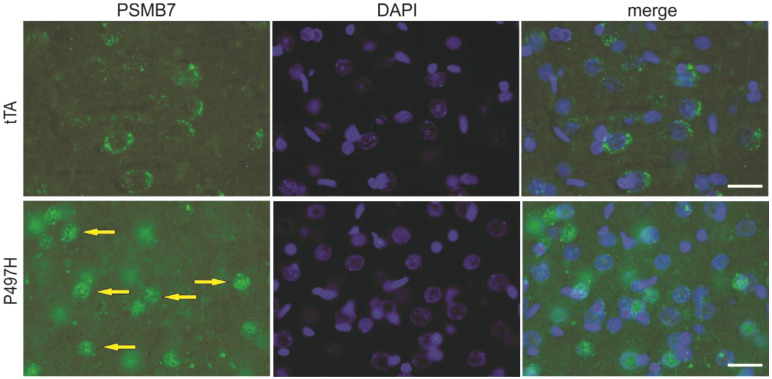
Proteasome subunit PSMB7 localization in mutant UBQLN2 rats. Immunofluorescence staining showed that PSMB7 was mislocalized to nuclei in the frontal cortex sections from 40-week-old mutant UBQLN2 rats (yellow arrows). Scale bars 30 μm.

Increasingly, evidence has implicated ALS/FTD-linked UBQLN2 mutations to be the cause of proteasomal impairment [4,10,16,26], and the formation of protein inclusions has been demonstrated in mutant UBQLN2 rats [6,26]. To examine whether mutant UBQLN2 leads to mislocalization of proteasome subunits, tissue lysates from rat frontal cortex were separated into nuclear and cytoplasmic fractions. In line with our previous study [6], we observed that both human and rat UBQLN2 proteins were enriched in the nuclear fraction of mutant UBQLN2 rats at 40 weeks of age instead of 4 weeks of age (Figure 3). In addition to the mislocalization of the UBQLN2 protein, a variety of proteasome subunits (e.g., PSMA1, PSMB7, Rpt1, and Rpn1) were also translocated to the nucleus (Figure 3). To confirm these findings, immunofluorescence staining was used to assess tissue localization, and nuclear colocalization of PSMB7 was observed (Figure 4). These data suggest that mutant UBQLN2 promote the translocation of proteasome subunits to rat nuclei.

### 2.4. In Vitro Dissociation of the 26S Proteasome by Mutant UBQLN2

**Figure 5:**
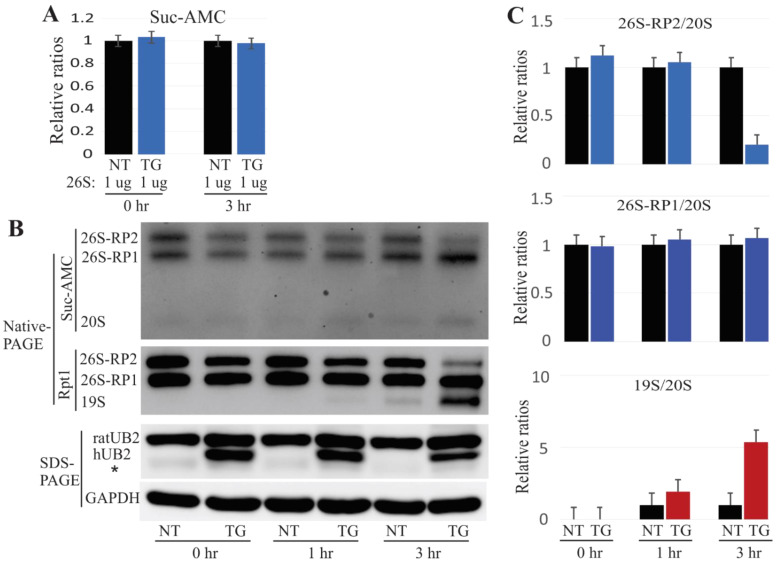
In vitro dissociation of the 26S proteasome by mutant UBQLN2. (**A**). The overall activity of proteasomes was measured using the sensitive fluorogenic substrate Suc-AMC. The data are reported as the mean ± standard deviation (*N* = 3) and showed no significant difference. (**B**). Purified 26S proteasome was incubated with native protein lysates for the times (hr) indicated, followed by in-gel assay assessment using the proteasome substrate Suc-AMC and immunoblot analysis using indicated antibodies for either NativePAGE or SDS-PAGE. (**C**). Based on the Panel B data, the relative ratios of the 26S or the 19S to the 20S proteasome were calculated by Image-J software (the NT rat value was designated as 1). This analysis showed that mutant UBQLN2 led to reduction of 26S proteasome but a 19S proteasome increase. NT: non-transgenic rats; TG: mutant UBQLN2-P497H rats; 26S-RP1/26S-RP2: 26S containing one or two regulatory subunits.

Several in vitro and in vivo reports have implicated mutant UBQLN2 in impaired proteasome functions [4,10,16,26]. In the present UBQLN2 transgenic rats, 26S proteasome integrity was impaired as early as four weeks of age (Figure 1). To examine whether the mutant UBQLN2 was responsible for proteasome dissociation, we incubated 4 µg of purified 26S proteasome with 30 µg of native protein lysate from 4-week-old rats at 37 °C for different amounts of time. We then assessed proteasome activity in these samples using the Suc-AMC substrate, and observed that the overall proteasome activity was unchanged (Figure 5A). However, the in-gel assay revealed that proteasome 26S-RP2 (containing two regulatory particles) decreased dramatically after three hours of incubation. Similar results were obtained by immunoblot analysis using the Rpt1 antibody, which showed that 19S was slightly elevated at the one-hour incubation time point but significantly elevated greatly at the three-hour time point (Figure 5B,C). These results were consistent with our previous finding that impairment of proteasome assembly occurred prior to compromised proteasome activity (Figure 1), and suggest that the ALS/FTD-linked UBQLN2 mutation dissociates 26S proteasome into 20S and 19S particles.

### 2.5. ALS/FTD-Linked UBQLN2 Mutations Did Not Affect Proteasome Function in HEK-293T Cells

**Figure 6:**
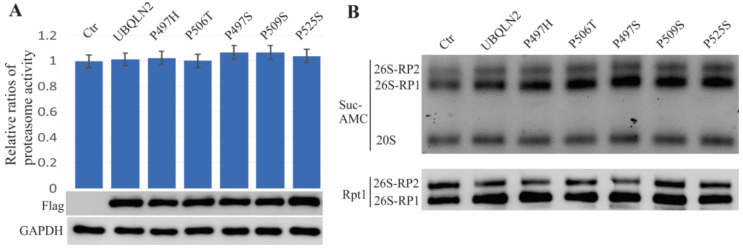
Mutant UBQLN2 does not lead to proteasome impairment in HEK-293T cells. Cells were transfected with either wild-type UBQLN2 or with ALS/FTD-linked UBQLN2 mutants and harvested cells in 48 h after transfection. (**A**). The chart revealed that the overall activity of proteasomes was measured using the Suc-AMC substrate, and the immunoblot assay showed similar expression levels for the wild-type and mutant UBQLN2 transfections. (**B**). The in-gel assay assessment revealed that 26S proteasome assembly was not impaired in mutant UBQLN2 transfected cells.

To further examine the effects of ALS/FTD-linked UBQLN2 mutations on proteasome functions, wild-type or mutant UBQLN2 (P497H, P497S, P506T, P509S, or P525S) were overexpressed in HEK-293T cells. We harvested cells at 48 h of after the transfection, and three fluorogenic substrates (Suc-AMC, Boc-AMC, or Z-AMC) were used to assay proteasome activity. None of the assays using these three substrates showed alterations, illustrated only by the Suc-AMC data (Figure 6A). The five mutations used for these assays were first reported by Deng et al. [1]. We then resolved the native protein lysates with Native-PAGE but found no alterations detected either by in-gel assay or by immunoblot using the Rpt1 antibody (Figure 6B). These results are consistent with a previous report that proteasome activity (chymotrypsin-like activity) was not altered by these five UBQLN2 mutations in Hela cells [16].

## 3. Discussion

The UBQLN2 protein shuttles between the nucleus and cytoplasm and is associated with protein degradation via both proteasomes and autophagy [12,14,21]. Proteasome impairment has been implicated in the pathogenesis of UBQLN2-associated ALS/FTD [1,6,16]. Here, we demonstrated that an ALS/FTD-linked UBQLN2 mutant impaired assembly of the 26S proteasome in rats and also dissociated the 26S proteasome in vitro.

Increasingly, ALS/FTD-linked UBQLN2 mutations have been shown to cause defective protein degradation by [1,15,16,27]. Combined with our recent report that a UBQLN2 mutant was responsible for the accumulation of the proteasome subunit Rpt1 [26], these findings suggest that UBQLN2 mutations can lead to impairment of proteasome functions. Consistent with these reports, we found that the 26S proteasome decreased dramatically in rats at the age of 4 weeks, even though the overall proteasome activity in this model system was unchanged until the age of 20 weeks, at which time proteasome impairment deteriorated in concert with disease progression. These findings further support the idea that mutant UBQLN2 impairs proteasomes. We previously reported that mutant UBQLN2 caused neuronal loss to occur in rats at the age of 130 days instead of 50 days, and UBQLN2 depletion did not cause any detectable alteration at the age of 280 days [6]. Consistently, we observed no differences for both overall proteasome activity and proteasome assembly in UBQLN2-depleted rats as old as 18 months, suggesting that impaired proteasome assembly due to the UBQLN2 mutation precedes their neuronal loss and providing further evidence for a gain-of-toxicity function that causes neurodegeneration.

Protein aggregation is one of the prominent features in Ubqln2-linked ALS/FTD [1], and rodent models overexpressing mutant UBQLN2 also demonstrated this [4,6,7]. The aggregated UBQLN2 protein was found to accumulate in both the cytoplasm and nuclei in our mutant UBQLN2 rats [6]. In these rodent models, mutant UBQLN2 entraps these proteasome components, and thus may prevent these subunits from assembly into functional proteasomes and lose their ability to perform normal functions. Similar to previous reports, we found that a variety of proteasome components, including 20S core particles (PSMA1 and PSMB7), 19S regulatory particles (Rpt1 and Rpn1), and mutant UBQLN2 protein were enriched in nuclei of old mutant UBQLN2 rats compared to those from one-month-old rats, indicating that this ALS/FTD-linked UBQLN2 mutant caused their mislocalization and that these abnormal accumulations were likely responsible for the age-dependent proteasome impairment.

Protein mislocalization is observed in our rat models that overexpress mutant UBQLN2 either in brain neurons [6] or in spinal motor neurons [5] or in astrocytes [5]. It is also observed in several mouse models reported by different groups. Viral expression of mutant UBQLN2 in mice showed that WT UBQLN2 was mainly cytoplasmic and diffuse, and mutant UBQLN2 exhibited prominent nuclear localization [28]. UBQLN2 driven by the neuron-specific Thy1.2 expression cassette in mice showed that UBQLN2 mutants rather than wild-type form led to cytoplasmic TDP-43 aggregation [7]. In addition, UBQLN2 driven by the mouse prion promoter in mice revealed that P506T-UBQLN2 resulted in mislocalization of TDP-43 and sequestration of proteasome subunits [8,29]. Taken together, these reports indicate that protein mislocalization is a hallmark in ALS/FTD-linked with UBQLN2 mutations, at least in rodent models.

UBQLN2 is a shuttle protein that facilitates the proteasome to degrade ubiquitinated proteins [17]. Fully functional proteasomes consist of one 20S core particle and either one or two 19S regulatory particles [18,19,20]. Compared to the 20S particle, the 26S proteasome is more important for protein homeostasis and is therefore more relevant to disease progression, as shown by our in vivo result that proteasome assembly is impaired at early stages of the disease in mutant UBQLN2 rats. Consistently, we obtained similar results from the in vitro proteasome dissociation assay: 26S-RP2 was dramatically reduced but 19S was greatly increased after three hours of coincubation with purified 26S and mutant UBQLN2 native protein lysates. The in vitro assay results suggest that the UBQLN2 mutation is responsible for dissociating the 26S proteasome into 20S and 19S particles, further evidence that mutant UBQLN2 impaired proteasome integrity.

Consistent with the present findings in HEK-293T cells, a recent study reported no dysfunction of the proteasome examined by the Suc-AMC substrate in Hela cells that overexpressed ALS/FTD-linked UBQLN2 mutants (P497H, P497S, P506T, P509S, or P525S) [16]. In addition, the present in-gel assay results also showed no impairment of proteasome assembly. There maybe two reasons for discrepancies between the in vitro and in vivo results. The first is that mutant UBQLN2 was only expressed in neurons in our in vivo rat model, and neurons are known to be more vulnerable to damage compared to other cells. The other potential reason is that both ALS and FTD are age-dependent disorders characterized by the slow and progressive loss of neurons in the central nervous system [30]. Therefore, the temporary over-expression of ALS/FTD-linked UBQLN2 mutants in HEK-293T or Hela cells may not have been sufficient to cause any overt proteasome alterations.

Taken together, the present data suggest that impairment of proteasome integrity occurs during early stages of the disease process associated with UBQLN2 mutations, and that these early-stage changes are more relevant to disease progression compared to late-stage alterations, at least in rat models. These novel findings may therefore contribute to a novel strategy for early disease diagnosis and to a promising therapeutic approach that targets proteasome integrity for these incurable disorders.

## 4. Materials and Methods

### 4.1. Genotyping of Transgenic Rats

All rats were maintained on Sprague-Dawley background. As characterized previously [6], CaMKα2-tTA transgenic rats [31] were crossed with TRE-UBQLN2^P497H^ transgenic rats to generate bigenic rats. Breeding rats were fed doxycycline (DOX) (50 μg/mL) (Sigma, St Louis, MI, USA) in drinking water during embryonic development. The DOX was removed from their drinking water at birth to induce the expression of mutant UBQLN2^P497H^ transgene. The followed primers were used for the identification of transgenic rats: CaMKα2-tTA (5′-GAAGCCCTATTTCTAGCTGTC-3′ and 5′-CTAAATGCCACAGGGTCTTGC-3′) and TRE-UBQLN^P497H^ (5′-AGGATCATAATCAGCCATACCAC-3′ and 5′-CTGCACCTAGTGAAACCACGA-3′). The UBQLN2 knock-out rats were identified using the PCR and the primers: (5′-CAGCAGTTCAAGGAAGCGAT-3′ and 5′-CGTGGTCGTCGTGCTCGTTC-3′).

### 4.2. Plasmid Construction and Cell Culture

Plasmids were constructed as previously described [31], and a 3× FLAG tag was fused to the C-terminal of human UBQLN2. HEK-293T cells were obtained from American Type Culture Collection (ATCC, Manassas, VA, USA) and were cultured in Dulbecco’s modified Eagle medium supplemented with 10% fetal bovine serum (FBS) and antibiotics (ampicillin and streptomycin). Plasmids were transfected using lipofectamine-2000 according to the manufacturer’s instructions (Life Technologies, Grand Island, NY, USA).

### 4.3. Cell Fractionation

Rat tissues were fractionated with a modified protocol [32]. Tissues were homogenized in ice-cold PBS and then centrifuged (10 min, 13,000 rpm in a benchtop centrifuge, at 4 °C), discarded the supernatant; The pellets were resuspended in 1 mL of Buffer A (50 mM K-HEPES (pH 7.5), 10 mM KCl, 1.5 mM MgCl_2_, 1 mM EDTA, 1 mM EGTA, and 0.5% Triton X-100 plus complete protease inhibitors). Of this cell suspension 200 μL was then sonicated and centrifuged at 13,000 rpm for 5 min, kept the supernatant representing the whole tissue lysate. The remaining 800 μL of the cell suspension was incubated on ice for 10 min and then centrifuged at 13,000 rpm for 5 min to obtain a cytoplasmic supernatant and a nuclear pellet. The nuclear pellet was washed with Buffer A and resuspended in 800 μL Buffer B (Buffer A + 1 M sucrose), and then centrifuged for 10 min at 6000 rpm. All pellets were resuspended in 300 μL Buffer C (50 mM K-HEPES (pH 7.5), 400 mM KCl, 1.5 mM MgCl_2_, 1 mM EDTA, 1 mM EGTA, and 10% glycerol plus complete protease inhibitors), sonicated, and then centrifuged at 6000 rpm for 10 min with the supernatants as the nuclear fractions.

### 4.4. SDS-PAGE Electrophoresis and Fluorescence Staining

Total protein lysates from rat tissues were extracted with 1× RIPA buffer and separated via SDS-PAGE for immunoblotting as described previously [33]. The resolved proteins were transferred onto nitrocellulose membranes and detected using the following primary antibodies: mouse anti-Ubqln2 (Abnova; Taipei, Taiwan), mouse anti-Flag (Sigma, St Louis, MI, USA), and the followed antibodies were purchased form Proteintech (Rosemont, IL, USA): rabbit anti-P62, rabbit anti-PSMA1, rabbit anti-PSMB7, rabbit anti-Rpt1, rabbit anti-Rpn1, rabbit anti-Histone 3, and mouse anti-GAPDH. Rat brains were fixed in 4% paraformaldehyde and then cryopreserved using 30% sucrose prior to cryostat sections. Tissues were sectioned transversely (20 μm) and stained using the rabbit anti-PSMB7.

### 4.5. Proteasome Activity Assays and Native-PAGE Electrophoresis

Native protein lysates were extracted from both rat forebrains and transfected cells using lysis buffer (50 mM Tris.HCl (pH 7.5), 5 mM MgCl_2_, 50 mM NaCl, 0.5 mM EDTA, 10% glycerol plus 4 mM ATP, 1 mM DTT, and 1× protease inhibitor). Proteasome activity in total tissue lysates was measured using proteasome assay buffer (50 mM Tris.HCl (pH 7.5), 5 mM MgCl_2_ plus 1 mM ATP, and 1 mM DTT) containing the fluorogenic substrate Boc-LRR-AMC (Boc-AMC), Suc-LLVY-AMC (Suc-AMC), or Z-LLE-AMC (Z-AMC) (purchased from UBPBIO.COM) (Aurora, CO, USA) for determining trypsin-, chymotrypsin-, or caspase-like proteasome activity, respectively. Of total lysates 50 µg were incubated with 50 µM proteasome substrate for 30 min at 37 °C. Then the fluorescence intensity was measured using a fluorescent plate reader (380 nm excitation/460 nm emission).

NativePAGE™ 3–12% Bis-Tris protein gels were used for the in-gel peptidase activity assays. Of total protein lysates 50 µg were first resolved at 100 V for 1 h and then at 150 V for an additional 2 h in a cold room. The gels were then incubated in proteasome assay buffer containing 50 µM Suc-AMC for 15 min at 37 °C. AMC release was visualized under UV light.

### 4.6. Proteasome Dissociation Assay

The dissociation assay was performed using proteasome assay buffer (50 mM Tris.HCl (pH 7.5), 5 mM MgCl_2_ plus 1 mM ATP, and 1 mM DTT). Purified 26S proteasome (4 µg) was incubated in this buffer with 40 µg of total protein lysates from rat frontal cortex in 50 µL total volume, and collected 10 µL sample for Native-PAGE analysis at the indicated time points and stopped the reaction by freezing these reactions on dry ice, followed by the in-gel assay, and for the immunoblot analysis using the indicated antibodies. The overall proteasome activity was measured immediately using 5 µL samples at each time point.

### 4.7. Statistical Analysis

All data shown are representative and all experiments were repeated at least three times independently. Statistical analysis was carried out using one-way ANOVA test. *p* < 0.05 was considered statistically significant.

## Data Availability

No applicable.
